# News and views

**DOI:** 10.1111/iwj.13882

**Published:** 2022-07-15

## 1 HEALIVA ACQUIRES TWO WOUND‐HEALING CELL THERAPY ASSETS FROM SMITH+NEPHEW

Healiva, a patient‐centric company delivering life‐enhancing precision medicine for patients with chronic and acute wounds, announced the acquisition of two innovative cell therapy assets from Smith+Nephew, the global medical technology business. The acquisition enables Healiva to establish one of the world's broadest portfolios of affordable, personalised, end‐to‐end wound care consisting of enzyme technology, autologous & allogenic cell therapies, and medical devices. Financial details have not been disclosed.

The first asset, EpiDex, is an autologous epidermal equivalent that provides a surgery‐free approach to healing chronic venous leg ulcers (VLUs). Having previously been approved for the Swiss market, Healiva's acquisition of the complete product dossier should enable the rapid market re‐launch of EpiDex.

The second asset, now named healiva002, is an off‐the‐shelf allogeneic cell therapy that has previously demonstrated clinical efficacy in healing VLUs that are resistant to standard treatments.

Today, the standard treatment for VLUs and DFUs requires multiple procedures which come with lengthy recovery times. In severe chronic cases, surgery is required. Crucially, less than 50% of such wounds will heal following surgery and those that do not heal will continue to worsen, potentially leading to amputation or, in severe cases, mortality. In the absence of similar market offerings, EpiDex fills an important niche by promoting healing even in the most severe wounds, with a closure rate equivalent to surgical methods, while being both more cost‐effective and significantly preferable for patients.

About Healiva.

Founded in 2020 by Bioseutica BV and Dr. Priyanka Dutta‐Passecker, Healiva is a patient‐centric biotech company delivering life‐enhancing precision medicine for patients with chronic and acute wounds. Healiva creates tailored wound solutions through a multi‐pronged approach that combines enzyme technology, medical devices, and cell therapies. Cell therapy describes a process in which viable cells are introduced into the body to produce medicinal effects; cells may be derived from a cell bank (allogeneic cell therapy) or be patient‐derived cells converted into the desired cell type or tissue in a lab (autologous cell therapy). Through the acquisition, development and commercialization of these assets, Healiva enters the billion‐dollar wound‐care market. To find out more, visit healiva.com.

## 2 KERECIS

Kerecis, the company pioneering the use of fish skin and fatty acids in cellular therapy, tissue regeneration and protection, announced MariGen Expanse, the newest addition to Kerecis' product offerings for chronic wound management. The announcement was made at the Symposium for Advanced Wound Care (SAWC) meeting, which is taking place in the Phoenix Convention Center from April 6 to 10, 2022. Kerecis is exhibiting at booth 325.

This pre‐meshed, intact‐fish‐skin graft supports healing and tissue regeneration in large chronic wounds especially wounds 100 square centimetres (15.5 square inches) and larger that are treated in an out‐patient care setting. MariGen Expanse is an extension of Kerecis' MariGen product line, which has been used to successfully treat tens of thousands of patients, prevented multiple amputations and improved the quality of life for many people around the world.

MariGen Expanse is available now in two pre‐meshed sizes: 7 × 8 centimetres (3 × 3 inches) and 8 × 10 centimetres (3 × 4 inches). Due to its 2:1 meshing, the product can be stretched to cover up to 101 and 144 square centimetres (15 and 22 square inches) respectively.

Kerecis products are based on fatty‐acid‐rich, intact fish skin that, when grafted onto damaged human tissue, recruits the body's own cells and ultimately is converted into living tissue. Because no disease‐transfer risk exists between cold‐water fish and humans, the Kerecis fish skin is only gently processed and retains its similarity to human tissue. The gentle processing preserves the skin's original three‐dimensional structure and maintains the skin's inherent natural strength, complexity and molecules (such as Omega3 and other fatty acids). Clinical studies have found that the Kerecis products heal wounds faster than other products.

In addition to the new MariGen product, the company offers three other product lines. GraftGuide addresses the management of burn injuries; SurgiClose and SurgiBind are designed to address surgical deficiencies.

### 2.1 About Kerecis

Kerecis develops products from fish skin and fatty acids for cellular therapy, tissue regeneration and protection. The Kerecis intact fish skin protects the body's tissues and enables it to regenerate tissues. The Kerecis sprayable topical and oral formulations protect the body from bacterial and viral infections.

Kerecis is the only approved manufacturer of medical devices containing intact fish skin globally. It is the fastest‐growing company and one of the top eight in the U.S. biologics‐skin and dermal‐substitute market, according to SmartTRAK Business Intelligence.

Kerecis is committed to the United Nations Sustainable Development Goals. The fish skin used in Kerecis products derives from wild and sustainable fish stock caught in pristine Icelandic waters and processed with 100% renewable energy in the town of Isafjordur, close to the Arctic Circle. For more information, visit https://www.kerecis.com.

## 3 AATRU MEDICAL

Aatru Medical, is pleased to announce a Latin America distribution agreement with Salus Biomedical, for the NPSIMS product. The product received recent FDA 510(k) clearance, and Aatru is in the process of finalising market introduction plans. Initial markets include Latin America, with U.S. and additional global markets to follow.

The patented NPSIMS utilises a safe and innovative solid‐state chemical reaction to create and apply negative pressure in the therapeutic range to closed incision sites for up to 7 days. The unique mode‐of‐action, which contains no moving parts or electromechanical components, delivers similar clinical performance to other available Negative Pressure Wound Therapy (NPWT) devices while providing substantial advantages in patient quality of life, ease of use, and price.

Several post‐operative wound complications are common following surgical procedures. Negative pressure wound therapy (NPWT) is well recognised for the management of open wounds, and in the last several years has been applied to closed surgical incisions. Compared with standard postoperative dressings, NPWT has been clinically shown to significantly reduce the rate of surgical site infection and seroma.

### 3.1 About Aatru medical

Aatru is a privately held medical device technology company focused on disrupting the surgical incision market with its FDA‐cleared NPSIMS, a simple, disposable, single‐use, patented, low‐cost NPWT device, uniquely designed to require no electromechanical pump, battery or canister. More information can be found on the Aatru Medical, LLC website: www.aatru.com

## 4 LIMFLOW

### 4.1 LimFlow completes enrollment in PROMISE II U.S. pivotal trial of breakthrough device designed to prevent amputations in no‐option patients with chronic limb‐threatening ischemia

LimFlow, a pioneer in the development of minimally‐invasive technology for the treatment of chronic limb‐threatening ischemia (CLTI), a severe form of peripheral artery disease (PAD), announced that it has completed patient enrollment in the PROMISE II pivotal trial of the LimFlow System. The LimFlow System is designed to re‐establish arterialization in deep veins for patients who have exhausted other methods and face major amputation of their lower limbs.

PROMISE II is a multi‐center, prospective, single‐arm study being conducted at multiple sites in the U.S. Using an adaptive statistical design, the study enrolled 105 no‐option CLTI patients. Endpoints include amputation‐free survival at 6 months, limb salvage and wound healing, and subjects will be followed out for 3 years. The no‐option patients treated in PROMISE II were determined by an independent physician committee to be no longer eligible for conventional endovascular or surgical therapy to treat CLTI.

LimFlow also announced the completion of enrollment in the CLariTI study of approximately 200 high‐risk and no‐option CLTI patients. The prospective, observational, multicenter CLariTI study will track the clinical progression of CLTI and incidence of death, amputation, and revascularization attempts in patients undergoing standard medical management for the disease over a one‐year period.

CLTI is the most severe form of PAD and often occurs in patients suffering from coronary artery disease, diabetes, obesity, high cholesterol and/or high blood pressure. Patients with CLTI often experience profound, chronic pain and develop festering wounds or infections that lead to major limb amputation, an event closely associated with increased mortality and reduced quality of life. To relieve the symptoms of CLTI, patients today are treated primarily with angioplasty or open bypass surgery. In many late‐stage patients, however, neither option is feasible due to extensive disease in the target arteries or other anatomical constraints.

About LimFlow and the LimFlow System.

LimFlow is a private, venture‐backed medical device company transforming the treatment of chronic limb‐threatening ischemia, a growing clinical need in the face of the prevalence of diabetes, heart disease, kidney disease and an aging population.

When all other therapeutic options have been exhausted and a CLTI patient is facing major amputation, the minimally invasive LimFlow system is designed to bypass blocked arteries in the leg and deliver oxygenated blood back into the foot via the veins. For many patients, restoring perfusion in the lower limbs resolves chronic pain, improves the quality of life, enables wound healing, and prevents major amputation.

For more information, visit www.limflow.com.

